# Distinct hepatic myeloid and lymphoid cell repertoires are associated with susceptibility and resistance to *Ascaris* infection

**DOI:** 10.1017/S0031182021000020

**Published:** 2021-04

**Authors:** Gwendoline Deslyper, Dearbhla M. Murphy, Oluyomi A. Sowemimo, Celia V. Holland, Derek G. Doherty

**Affiliations:** 1 Department of Zoology, School of Natural Sciences, Trinity College Dublin, Dublin, Ireland; 2 Department of Immunology, School of Medicine, Trinity College Dublin, Dublin, Ireland; 3 Department of Zoology, Obafemi Awolowo University, Ile-Ife, Nigeria

**Keywords:** *Ascaris suum*, *Ascaris lumbricoides*, flow cytometry, liver, mouse model, resistance, susceptibility

## Abstract

The soil-transmitted helminth *Ascaris lumbricoides* infects ~800 million people worldwide. Some people are heavily infected, harbouring many worms, whereas others are only lightly infected. The mechanisms behind this difference are unknown. We used a mouse model of hepatic resistance to *Ascaris*, with C57BL/6J mice as a model for heavy infection and CBA/Ca mice as a model for light infection. The mice were infected with the porcine ascarid, *Ascaris suum* or the human ascarid, *A. lumbricoides* and immune cells in their livers and spleens were enumerated using flow cytometry. Compared to uninfected C57BL/6J mice, uninfected CBA/Ca mice had higher splenic CD4^+^ and *γδ* T cell counts and lower hepatic eosinophil, Kupffer cell and B cell counts. Infection with *A. suum* led to expansions of eosinophils, Kupffer cells, monocytes and dendritic cells in the livers of both mouse strains and depletions of hepatic natural killer (NK) cells in CBA/Ca mice only. Infection with *A. lumbricoides* led to expansions of hepatic eosinophils, monocytes and dendritic cells and depletions of CD8^+^, *αβ*, NK and NK T cells in CBA/Ca mice, but not in C57BL/6J mice where only monocytes expanded. Thus, susceptibility and resistance to *Ascaris* infection are governed, in part, by the hepatic immune system.

## Introduction

An estimated 800 million people are infected with the human roundworm *Ascaris lumbricoides* (Pullan *et al*., 2014). Despite this high number of infected individuals, ascariasis remains a neglected tropical disease (Deslyper and Holland, 2017; Hotez *et al*., 2020; World Health Organization, 2020). Intensity of infection is not evenly distributed among the population, where a small subset of the population carries the majority of the worm burden (Bethony *et al*., 2006). Furthermore, people regain similar worm burdens upon reinfection, even after several rounds of chemotherapy (Seo *et al*., 1979; Anderson and May, 1982; Croll *et al*., 1982; Elkins *et al*., 1986; Holland *et al*., 1989). This is known as predisposition and appears to be multifactorial in origin (Holland, 2009) with both long-term (host genetics and socio-economic status) and short-term (host-acquired immune system) factors involved (McCallum, 1990).

As heavy worm burden is associated with more severe symptoms (Croll and Ghadirian, 1981; Holland, 2009), it is important to understand the underlying molecular mechanisms associated with the observed predisposition. Because the early life cycle of the parasite includes internal organs of the host, it is necessary to use animal models (Holland *et al*., 2013). Building on an earlier study (Mitchell *et al*., 1976), our group (Lewis *et al*., 2006) developed a mouse model for resistance to *Ascaris suum* infection. We identified two mouse strains, one (C57BL/6J) as a model for susceptibility to heavy infection and another (CBA/Ca) as a model for resistance to *Ascaris* infection. Using this mouse model, the hepatic stage during larval migration was identified as the most likely time at which the observed differences between the two mouse strains in eventual lung larval burdens were generated (Lewis *et al*., 2007; Dold *et al*., 2010). We subsequently used this mouse model to investigate the liver proteomes of the relatively resistant (CBA/Ca) and relatively susceptible (C57BL/6J) mouse strains, infected with *A. suum* (Deslyper *et al*., 2016; Deslyper *et al*., 2019*b*). We found intrinsic differences between the two mouse strains at the level of oxidative phosphorylation at days 4 and 7 post infection (p.i.) and at the level of the immune response proteins at day 7. The relatively resistant strain had a higher abundance of proteins associated with complement activation, whereas the relatively susceptible strain had a higher abundance of proteins associated with complement inhibition. These mouse models were also found to be suitable for infection with the human ascarid, *A. lumbricoides* (Deslyper *et al*., 2020).

The liver has special immunological properties. It receives blood directly from the gut *via* the hepatic portal vein. This blood carries with it antigens from both gut commensals and dietary products (Doherty, 2016). Because these antigens could cause unwanted chronic inflammatory responses, the hepatic immune system favours tolerance over immunity, which is mediated by specialized liver-resident antigen-presenting cells (Thomson and Knolle, 2010; Crispe, 2011). This feature makes the liver a potentially ideal organ for several parasites, including *Ascaris*, to incorporate in their migratory path, as a safe refuge and hence for immune evasion (Deslyper *et al*., 2019*a*).

To our knowledge, little research has been performed on the immune response in the liver to *Ascaris* infection. The only evidence of the immune response in the liver is the presence of white spots which have been observed in *A. suum*-infected pigs (Ronéus, 1966), *A. lumbricoides*-infected humans (Javid *et al*., 1999) and *A. suum*-infected mice (Dold *et al*., 2010). In the current study, we performed flow cytometry on spleen and liver samples from susceptible and resistant mice before and after infection with *A. suum* and *A. lumbricoides*. We selected day 7 p.i., because at this time point we previously found evidence for an altered immune response between the two mouse strains during *A. suum* infection (Deslyper *et al*., 2019*b*).

## Materials and methods

### Parasite eggs

The *A. lumbricoides* eggs were extracted from adult worms, which were obtained from dewormed children in Ile-Ife, Nigeria, using pyrantel pamoate. The adult worms were transported on ice in 4% formalin. They were dissected upon arrival and the uteri were mechanically broken up and sieved (425 *μ*m). The eggs were placed in 0.05 m H_2_SO_4_ (Aldrich) in culture flasks with a ventilated cap for embryonation of the eggs. The flasks were stored at 26°C and oxygenated twice per week.

The *A. suum* eggs were kindly donated by Dr Johnny Vlaminck (Ghent University). These were shipped in a water solution, stored at 26°C in 0.05 m H_2_SO_4_ and oxygenated twice per week.

### Infection of mice with *Ascaris* eggs

Fifteen male mice of both CBA/Ca OlaHsd (Envigo, UK) and C57BL/6J OlaHsd (Comparative Medicine Unit, Trinity College Dublin) were purchased; all mice were 8 weeks old at the time of the experiment. Five mice of each strain received oral gavage (Instech, FTP-20-38-50, USA) with either 1000 eggs of *A. suum*, 1000 eggs of *A. lumbricoides* or 100 *μ*L 0.05 m of H_2_SO_4_. The mice were culled at day 7 p.i. and the livers and spleens removed for flow cytometric analysis of immune cells. Additionally, the lungs were removed for larval counts using the modified Baermann method (Lewis *et al*., 2006).

### Larval recovery and enumeration for the lungs

After 24 h, the resulting samples from the Baermann method were centrifuged at 1389 ***g*** for 5 min. The supernatants were removed and 70% ethanol was added (50% v/v). Subsequently, the larvae were counted in 1 mL of sample solution, using a nematode counting chamber (Chalex Corporation, Park City, UT, USA).

## Retrieval of spleen and liver immune cells

After dissection, livers and spleens were kept in ice cold phosphate-buffered saline (PBS). Both organs were mechanically minced using sterile scalpels and sieved through a 70 *μ*m-gauge mesh in complete RPMI medium (cRPMI) (RPMI GlutaMAX™ supplemented with 1.25 mm HEPES and 10% foetal bovine serum, pH = 7.4). This resulted in single suspensions of spleen cells, of which the majority are circulating blood cells.

Since the liver contains resident, non-circulating immune cells embedded in a network of sinusoids which traverse the parenchymal tissue (Doherty, 2016), the immune cells must first be enzymatically extracted and separated from the parenchymal cells. The liver cell suspensions were suspended in 50 mL cRPMI and centrifuged for 1 min at 60 ***g*** without brake to remove undissociated tissue. The top 45 mL was removed and centrifuged again at 530 ***g*** for 10 min at 4°C. The resulting pellet was resuspended in 10 mL of digestion buffer (0.2 g L^−1^ collagenase from *Clostridium histolyticum* (Sigma-Aldrich) and 0.02 g L^−1^ DNase I (Sigma-Aldrich)) and incubated at 37°C for 30 min while shaking. Next, 30 mL PBS was added and left to rest on ice for 5 min before centrifugation at 528 ***g*** for 10 min at 4°C. The pellet was resuspended in PBS and layered over Lymphoprep™ (STEMCELL Technologies) and centrifuged at 400 ***g*** for 25 min without brake. The buffy coat layer, containing mononuclear cells (MNCs), was removed and kept aside. The pellet, containing erythrocytes and polymorphonuclear cells (PMNs), was incubated for 5 min at room temperature in red cell lysis buffer (0.1 mm EDTA, 155 mm NH_4_Cl, 10 mm KHCO_3_). Both MNC and PMN were centrifuged for 8 min at 480 ***g*** and the pellet resuspended in PBS and counted.

## Antibodies and flow cytometry

**Fig. 1.**
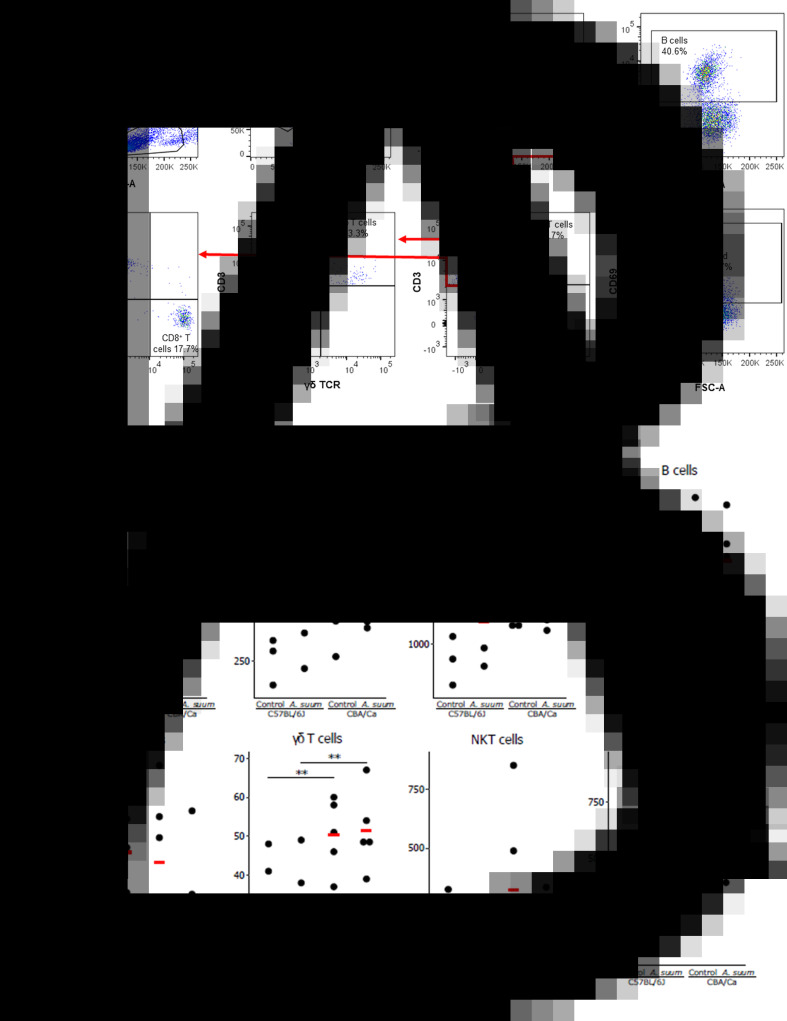
Lymphoid cell subtype numbers in the spleens of uninfected and *Ascaris suum*-infected C57BL/6J and CBA/Ca mice. (A) Gating strategy for the definition of lymphoid cell populations in spleens and livers. Following flow cytometric acquisition of MNCs, an electronic gate was placed on the lymphocytes based on forward and side scatter areas (FSC-A *vs* SSC-A) followed by gating of singlets (FSC-A *vs* FSC-H). Next, the live cells were gated upon in a dot plot of FSC-A *vs* dead cell stain (DCS). From these live cells, the activated cells were identified as CD69^+^ cells, T cells were identified as CD3^+^ cells and NKT cells were identified as CD3^+^ NK1.1^+^ cells. B cells were identified as CD19^+^ cells. *αβ* T cells (CD3^+^ and TCR *γ*/*δ*^−^) and *γδ* T cells (CD3^+^ and TCR *γ*/*δ*^+^) were identified after gating on CD3^+^ NK1.1^−^ cells. Finally, the *αβ* T cells were used to identify CD4^+^ and CD8^+^ T cells. (B) Scatter plots showing the lymphoid cell subtype numbers in the spleens of uninfected and *A. suum*-infected C57BL/6J and CBA/Ca mice. The numbers of cells per whole spleen for the different cell types for each sample are shown. The means are indicated with the red horizontal bars. **P < 0.01.

Approximately 0.5 × 10^6^ liver and spleen cells were pelleted by centrifugation and stained with a dead cell stain (Flexible viability dye; eBioscience; diluted 1/1000 in PBS) for 15 min at room temperature in the dark. Cells were then washed in PBA buffer (PBS containing 1% bovine serum albumin and 0.02% sodium azide), blocked with FcR blocking reagent (Miltenyi Biotec) to prevent non-specific binding of the antibodies to Fc receptor-positive cells, and washed again. Next, the antibodies were added and incubated for 15 min at room temperature in the dark. The panel for staining lymphocytes (Table 1) consisted of the following antibodies: APC/Cy7-conjugated anti-mouse NK-1.1 (PK136), PerCP/Cy5.5-conjugated anti-mouse CD19 (1D3/CD19), APC-conjugated anti-mouse CD69 (H1.2F3), FITC-conjugated anti-mouse CD4 (GK1.5), PE/Cy7-conjugated anti-mouse CD8 (53–5.8), PE-conjugated anti-mouse TCR *γ*/*δ* (UC7-13D5) and Pacific Blue™-conjugated anti-mouse CD3*ε* (145-2C11). The panel for the myeloid cells (Table 2) consisted of the following antibodies: PerCP/Cyanine5.5-conjugated anti-mouse F4/80 (BM8), APC/Cyanine7-conjugated anti-mouse CD11c (N418), APC-conjugated anti-mouse CD170 (Siglec-F) (S17007L), PE-conjugated anti-mouse CD200R3 (Ba13), Pacific Blue™-conjugated anti-mouse CD45 (30-F11), FITC-conjugated anti-mouse CD317 (BST2, PDCA-1) (927) and PE/Cy7-conjugated anti-mouse/human CD11b (M1/70). All antibodies were purchased from BioLegend (San Diego, USA). After staining, the samples were washed, fixed with 1% paraformaldehyde, washed again and analysed on a Becton Dickinson FACSCanto II flow cytometer. Data were analysed using Flow Jo software (Tree Star). Gating strategies for the detection and enumeration of lymphoid and myeloid cells are shown in Figs 1 and 3. Absolute numbers of splenic cell subtypes (per whole spleen) were calculated from the proportions determined by flow cytometry and viable cell counts obtained by fluorescent microscopy. Relative counts of liver cells (per mL of liver lymphoid or myeloid cell extract) were similarly determined, but the numbers of cells per whole liver were not calculated because parenchymal cells were removed and then lymphoid and myeloid cells were separated by centrifugation with cell losses, making it impossible to accurately relate cell yields to initial cell numbers.

**Table 1.** Antibody panel used for detection of lymphocytes for both liver and spleen

	NK1.1	CD19	CD3	CD4	CD8	CD69	*γδ* TCR
NK cells	+		**−**				
B cells	+		**−**				
CD4^+^ T cells			+	+	**−**		
CD8^+^ T cells			+	**−**	+		
*γδ* T cells			+				+
*αβ* T cells			+				**−**
Activated cells						+	
NKT cells	+		+

**Table 2.** Antibody panel used for detection of liver myeloid cells

	CD45	F4/80	CD11b	CD11c	CD317	CD170	CD200R3
Kupffer cells	+	+	+/**−**			**−**	
Eosinophils	+	+	+			+	
Monocytes	+		+				
Myeloid dendritic cells	+			+	**−**		
Plasmacytoid dendritic cells	+			+	+		
Mast cells and basophils	+						+

### Statistical analysis

The flow cytometry data were overdispersed, therefore a negative binomial distribution was found to be most appropriate. The MASS package (Venables and Ripley, 2002) was used for the negative binomial (log link) on each cell type. The most parsimonious model, a negative binomial without interaction between mouse strain and infection status of the mouse, was considered the default model. This model was compared to an interaction model, with an interaction between the mouse strain and infection status of the mouse. If the difference in absolute values of the Akaike information criterion (AIC) of the models (*Δ_i_* = AIC*_i_* − AIC_min_) was greater than 2, then the interaction model was used (Burnham and Anderson, 2004). Post-hoc tests with a multivariate testing adjustment were performed using contrasts in the emmeans package (Lenth, 2019). Post-hoc tests were performed between mouse strains for each species.

## Results

### Lung larval counts in susceptible and resistant mice

The mean number of larvae recovered from the C57BL/6J strain was higher for both *A. suum* and *A. lumbricoides* infection, compared to the respective CBA/Ca-infected mice. The larval counts were: C57BL/6J infected with *A. suum*: 31 ± 37.3 (mean ± s.d.), C57BL/6J infected with *A. lumbricoides*: 3 ± 2.74, CBA/Ca infected with *A. suum*: 7 ± 8.37 and CBA/Ca infected with *A. lumbricoides*: 1 ± 2.24.

### Effect of *A. suum* infection on spleen and liver cell numbers and phenotypes

#### Spleen lymphoid cells

Infection with *A. suum* did not elicit a statistically significant change in the numbers of any of the investigated spleen cell populations, CD8^+^ T cells, CD4^+^ T cells, *αβ* T cells, B cells, natural killer (NK) cells, *γδ* T cells, NKT cells or activated cells (Fig. 1). However, an intrinsic difference between the two mouse strains was observed for CD4^+^ T cells (*z* ratio: 2.505, *P* < 0.05) and *γδ* T cells (*z* ratio: 5.644, *P* < 0.01). For these cell populations, there were significantly higher numbers in the CBA/Ca mouse strain, compared to the C57BL/6J mouse strain, both with and without infection.

#### Differences in liver lymphoid cell numbers are mainly between mouse strains

**Fig. 2.**
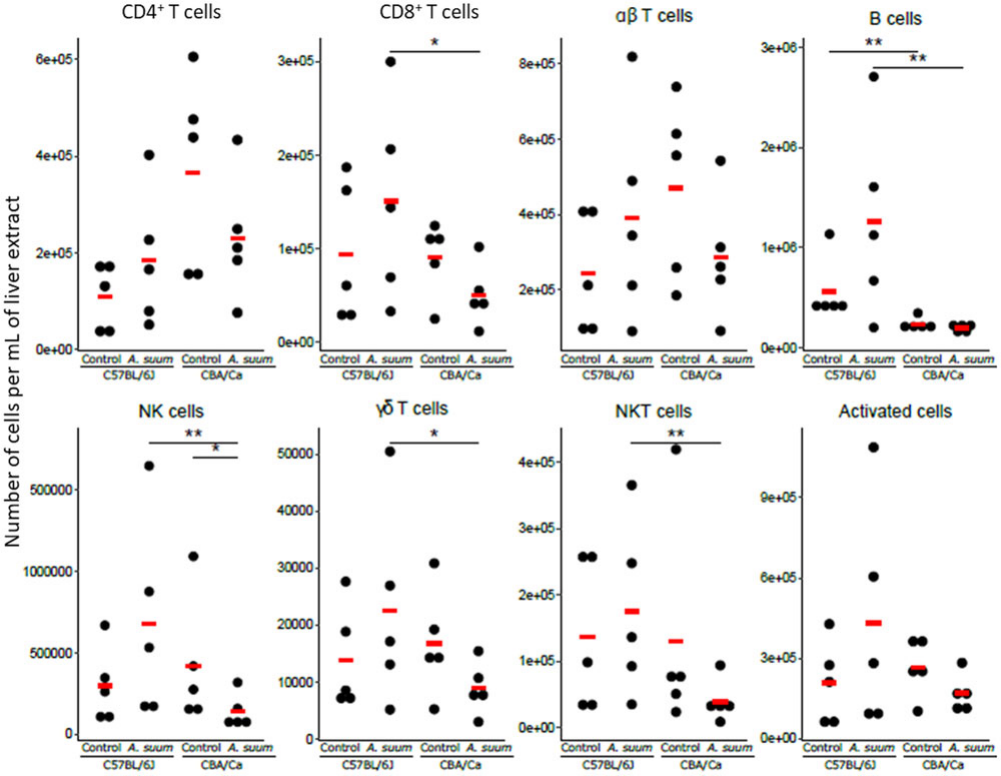
Lymphoid cell subtype numbers in the livers of uninfected and *A. suum*-infected C57BL/6J and CBA/Ca mice. The numbers of cells per mL of liver extract for the different cell types for each sample are shown. The means are indicated with the red horizontal bar. *P < 0.05; **P < 0.01.

For the liver lymphocytes (Fig. 2), the numbers of only one cell type, NK cells, was found to be statistically significantly different (*z* ratio: −2.766, *P* < 0.05) when comparing infected samples to their uninfected controls, and that occurred only for the CBA/Ca strain. Here, the control samples were found to have more NK cells than the infected samples.

An intrinsic difference between the two mouse strains, was found for hepatic B cell numbers (*z* ratio: −7.054, *P* < 0.01), with the C57BL/6J strain having a higher number of B cells compared to the CBA/Ca strain. Although the numbers of the other lymphoid cell populations tested were similar in both mouse strains, after infection with *A. suum*, the numbers of several cell types were found to be present in higher numbers in the livers of C57BL/6J mice compared with those of CBA/Ca mice. These are: CD8^+^ T cells (*z* ratio: −2.783, *P* < 0.05), B cells (*z* ratio: −7.054, *P* < 0.01), NK cells (*z* ratio: −4.003, *P* < 0.01), *γδ* T cells (*z* ratio: −2.761, *P* < 0.05) and NKT cells (*z* ratio: −3.293, *P* < 0.01).

#### Differences between hepatic myeloid cells are mainly between control and infection

**Fig. 3.**
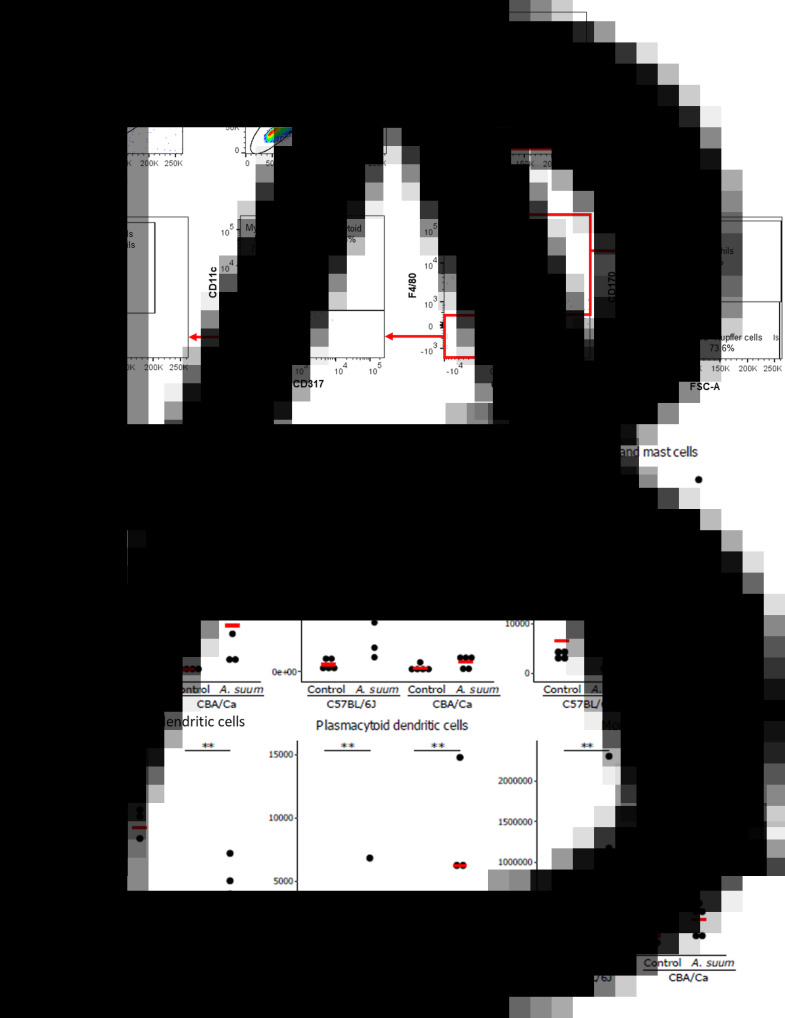
Myeloid cell subtypes in the livers of uninfected and *A. suum*-infected C57BL/6J and CBA/Ca mice. (A) Gating strategy for the definition of myeloid cell populations in livers. For analysis of myeloid cells, debris was eliminated by gating based on FSC-A *vs* SSC-A followed by isolation of singlets (FSC-A *vs* FSC-H) and live cells (FSC-A *vs* DCS). Next, the monocytes were identified by plotting CD11b against F4/80. Eosinophils and KCs were identified by gating on the F4/80^+^ CD11b^+^ cells and plotting FSC-A against CD170. Myeloid and plasmacytoid DC were identified from gated F4/80^−^ CD11b^−^ cells and plotting CD317 against CD11c. Finally, basophils and mast cells were identified by gating on CD11c^−^ and CD137^−^ cells and plotting FSC-A against CD200R3. Gates for spleen and liver lymphoid and myeloid cells were manually adjusted for every sample. (B) Scatter plots showing numbers of myeloid cell subtypes in the livers of uninfected and *A. suum*-infected C57BL/6J and CBA/Ca mice. The number of cells per mL of liver extract for the different cell types for each sample is shown. The means are indicated with the red horizontal bar. **P < 0.01.

Analysis of liver myeloid cells, revealed higher numbers of eosinophils (*z* ratio: −5.070, *P* < 0.01), and KCs (*z* ratio: −4.143, *P* < 0.01) in uninfected C57BL/6J compared to uninfected CBA/Ca livers (Fig. 3). For the eosinophils, this difference disappeared under *A. suum* infection. However, for the KCs this difference remained with the C57BL/6J mouse strain having a higher cell count (*z* ratio: −4.143, *P* < 0.01) than the CBA/Ca strain.

In both mouse strains, the numbers of eosinophils (C57BL/6J: *z* ratio: 3.309, CBA/Ca: *z* ratio: 7.095, both: *P* < 0.01), KC (C57BL/6J: *z* ratio: 4.648, CBA/Ca: *z* ratio: 4.648, both: *P* < 0.01), monocytes (C57BL/6J: *z* ratio: 4.962, CBA/Ca: *z* ratio: 4.962, both: *P* < 0.01) and dendritic cells (C57BL/6J: *z* ratio: 4.401, CBA/Ca: *z* ratio: 4.553, both: *P* < 0.01) were significantly higher in *A. suum*-infected livers compared to uninfected. In contrast, basophils and mast cells were found in similar numbers when comparing uninfected and infected mice.

### Effect of *A. lumbricoides* infection on spleen and liver cell numbers and phenotypes

#### Splenic lymphocytes

**Fig. 4.**
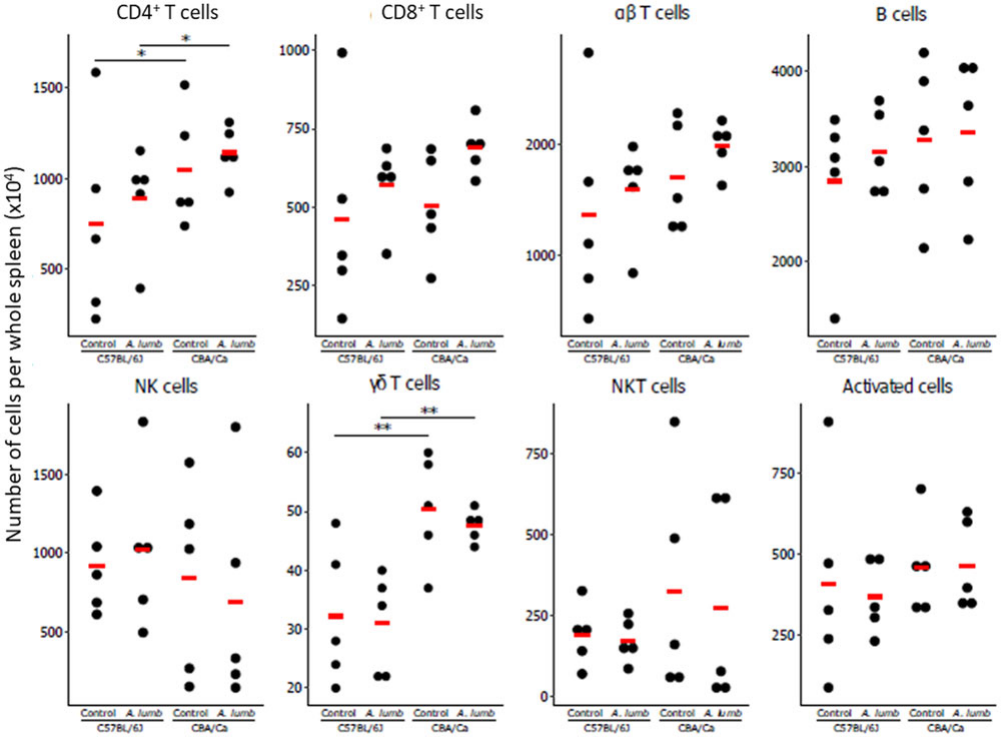
Lymphoid cell subtypes in the spleens of uninfected and *Ascaris lumbricoides*-infected C57BL/6J and CBA/Ca mice. The number of cells per whole spleen for the different cell types for each sample is shown. The means are indicated with the red horizontal bar. *P < 0.05; **P < 0.01.

When the immune cell composition in spleens of C57BL/6J and CBA/Ca mice infected with *A. lumbricoides* were examined, the frequencies of CD4^+^ T cells, CD8^+^ T cells, *αβ* T cells, B cells, NK cells, *γδ* T cells, NKT cells and activated T cells were found to be similar to those in uninfected mice. The only statistically significant differences (Fig. 4) found were higher numbers of CD4^+^ cells (*z* ratio: 2.505, *P* < 0.05) and *γδ* T cells (*z* ratio: 5.644, *P* < 0.01) in the CBA/Ca strain compared to the C57BL/6J strain. These higher numbers of CD4^+^ and *γδ* T cells were found in both uninfected and infected CBA/Ca mice.

#### Differences between liver lymphocytes

**Fig. 5.**
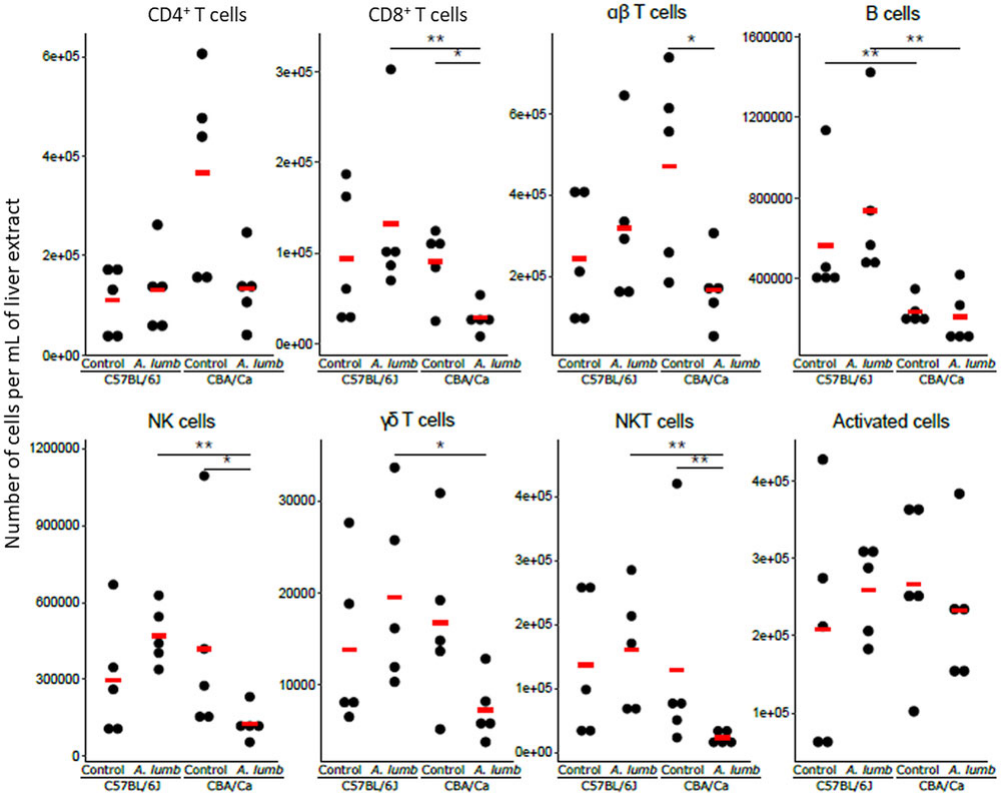
Lymphoid cell subtype numbers in the livers of uninfected and *A. lumbricoides*-infected C57BL/6J and CBA/Ca mice. The number of cells per mL of liver extract for the different cell types for each sample is shown. The means are indicated with the red horizontal bar. *P < 0.05; **P < 0.01.

Infection with *A. lumbricoides* did not lead to any statistically significant changes in numbers of hepatic lymphoid cells in C57BL/6J mice (Fig. 5). For the CBA/Ca strain, infection with *A. lumbricoides* led to significant decreases in the numbers of CD8^+^ T cells (*z* ratio: −2.939, *P* < 0.05), *αβ* T cells (*z* ratio: −2.895, *P* < 0.05), NK cells (*z* ratio: −3.049, *P* < 0.05) and NKT cells (*z* ratio: −3.783, *P* < 0.01). Compared to infected CBA/Ca mice, *A. lumbricoides*-infected C57BL/6J mice had higher numbers of CD8^+^ T cells (*z* ratio: −3.893, *P* < 0.01), B cells (*z* ratio: −7.054, *P* < 0.01), NK cells (*z* ratio: −3.345, *P* < 0.01), *γδ* T cells (*z* ratio: −2.918, *P* < 0.05) and NKT cells (*z* ratio: −4.258, *P* < 0.01).

#### Differences between liver myeloid cells

**Fig. 6.**
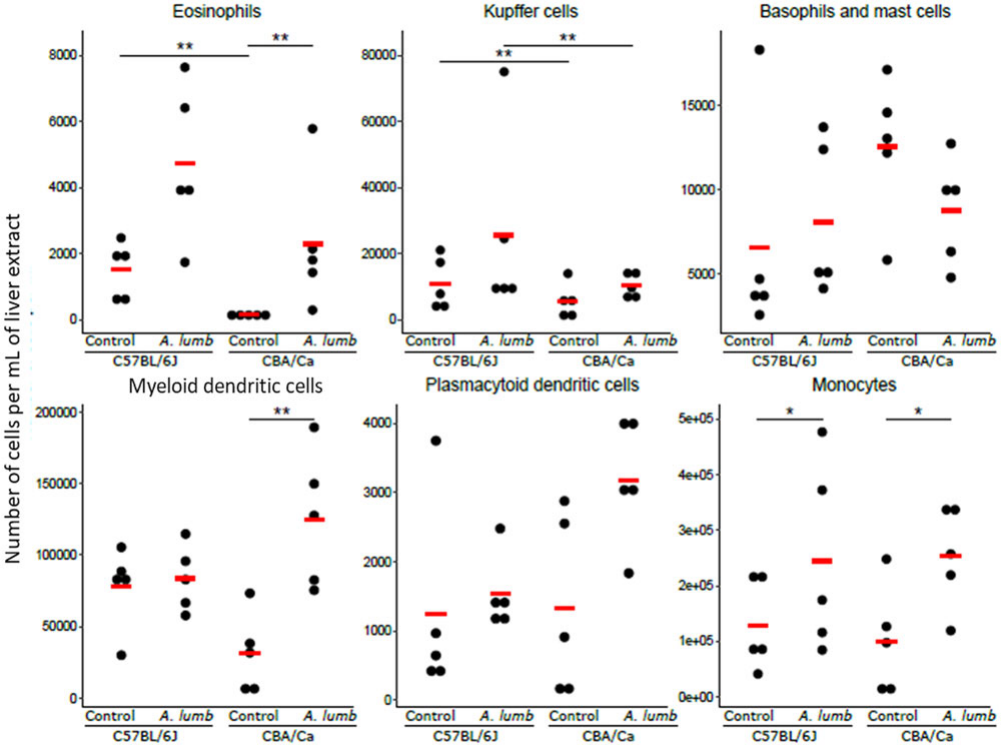
Myeloid cell subtype numbers in the livers of uninfected and *A. lumbricoides*-infected C57BL/6J and CBA/Ca mice. The number of cells per mL of liver extract for the different cell types for each sample is shown. The means are indicated with the red horizontal bar. *P < 0.05; **P < 0.01.

Analysis of hepatic myeloid cell numbers in control and *A. lumbricoides*-infected mice revealed that eosinophils (*z* ratio: 5.978, *P* < 0.01), DC (*z* ratio: 3.969, *P* < 0.01) and monocytes (*z* ratio: 2.483, *P* < 0.05) were expanded in infected CBA/Ca mice, whereas only monocytes (*z* ratio: 2.483, *P* < 0.05) were expanded in infected C57BL/6J mice. KCs were the only cell type whose numbers differed significantly in the two mouse strains after infection with *A. lumbricoides* (*z* ratio: −4.143, *P* < 0.01), with the C57BL/6J-infected samples having a higher number of cells compared to CBA/Ca-infected samples (Fig. 6).

## Discussion

In this study, we investigated the immune response in the liver during *Ascaris* infection, using a model of hepatic resistance, where one mouse strain (C57BL/6J) is relatively susceptible and another mouse strain (CBA/Ca) is relatively resistant to *Ascaris* infection (Lewis, 2006; Deslyper *et al*., 2020). The larval burdens in the lungs at day 7 p.i. support this model, with means of 7 and 31 *A. suum* larvae observed in the relatively resistant and relatively susceptible mouse strains, respectively, and 1 and 3 *A. lumbricoides* larvae observed in the resistant and susceptible strains.

The results of the current study indicate that the differences in susceptibility to *Ascaris* infection between the two mouse strains correlate with differences in the distributions of liver and spleen lymphoid cells. These differences are present both in uninfected and infected mice and are reflective of previous studies which found clear differences in the liver proteome in the two mouse strains, both uninfected and with *A. suum* infection (Deslyper *et al*., 2016; Deslyper *et al*., 2019*b*).

Our data indicate that infection with *Ascaris* is mainly associated with expansions or influxes of populations of myeloid cells in the livers. The relatively susceptible mouse strain had more eosinophils than the relatively resistant strain. However, under infection with either *A. suum* or *A. lumbricoides*, both mice showed expansions of eosinophils in their livers. Given the importance of eosinophils in parasite immunity in the lung and the gut, this is an expected finding (Enobe *et al*., 2006; Masure *et al*., 2013; Nogueira *et al*., 2016; Vlaminck *et al*., 2016; Weatherhead *et al*., 2018; Gazzinelli-Guimaraes *et al*., 2019). Eosinophils have been reported to expand in murine lungs during *A. suum* infection (Enobe *et al*., 2006; Nogueira *et al*., 2016; Weatherhead *et al*., 2018; Gazzinelli-Guimaraes *et al*., 2019) and are involved in reducing larval numbers (Gazzinelli-Guimaraes *et al*., 2019). In pigs, the presence of eosinophils has been linked to elimination of *Ascaris* in the gut (Masure *et al*., 2013; Vlaminck *et al*., 2016). Eosinophils also play roles in human ascarid infections, with expansions of these cells and elevated production of eosinophil cationic protein in putatively immune Nigerian children (McSharry *et al*., 1999) and chronically-infected Ecuadorian children (Reina Ortiz *et al*., 2011). Eosinophils can also paralyse *Schistosoma mansoni* (McLaren *et al*., 1984) and kill *Trypanosoma cruzi* and *Brugia malayi in vitro* (Hamann *et al*., 1990). A recent study found that immune serum-activated human macrophages coordinate with eosinophils to immobilize *A. suum* larvae (Coakley *et al*., 2020). The results of the current study further implicate hepatic eosinophils in the immune response to *A. suum* and *A. lumbricoides* in both mouse strains.

Monocytes and macrophages are also thought to contribute to immunity against *Ascaris*, being present in lung samples from *A. suum*-infected mice (Gazzinelli-Guimaraes *et al*., 2019) and capable of recognizing and responding to *A. suum in vitro* (Almeida *et al*., 2018; Coakley *et al*., 2020). We found that uninfected C57BL/6J and CBA/Ca had similar numbers of monocytes in their livers, and these cells were found in significantly higher numbers after infection with either *A. suum* or *A. lumbricoides*. However, the liver-resident macrophages, Kupffer cells, were present in higher numbers in the relatively susceptible C57BL/6J mice compared to the resistant CBA/Ca mice, both without and with infection by either *Ascaris* type. Under *A. suum* infection, however, there was a significant increase in Kupffer cell numbers for both mouse strains, but the relatively susceptible strain still had more of this cell type than the relatively resistant strain.

DCs are of interest in parasite infections because these cells are required for the induction of Th2 responses. Previous studies have found that *A. suum* has immunomodulatory effects on human DCs *in vitro* and that *A. suum* can upregulate a negative regulator of reactive oxygen species production (Favoretto *et al*., 2014; Midttun *et al*., 2018; Summan *et al*., 2018; Arora *et al*., 2020). We found that both myeloid and plasmacytoid DC were found in similar numbers in the livers of the two mouse strains, and that their numbers were significantly higher in *A. suum*-infected livers but only slightly higher in *A. lumbricoides*-infected livers.

Immune responses against parasites are controlled by a number of lymphoid cell types, in particular those that produce Th2 cytokines, such as interleukin (IL)-4, IL-5 and IL-13, which promote immunoglobulin E production and recruit and activate eosinophils and mast cells. We enumerated CD4^+^ and CD8^+^ T cells, T cells expressing *αβ* and *γδ* T cell receptors, NKT cells, B cells and NK cells in the spleens and livers of *A. suum* and *A. lumbricoides*-infected C57BL/6J and CBA/Ca mice. We found that the relatively resistant mouse strain had significantly more CD4^+^ and *γδ* T cells in their spleens than the relatively susceptible strain before infection and after infection with either ascarid species. While both CD4^+^ and *γδ* T cells can release Th2 cytokines, future studies are required to determine if the increased numbers of these cells in CBA/Ca mice compared to C57BL/6 mice reflect expansions of Th2 cells. A previous study, however, observed a reduction in CD4^+^ IL-4^+^ T cells in the spleen of *A. suum*-infected BALB/c mice (Gazzinelli-Guimarães *et al*., 2013), suggesting that the parasite may inhibit Th2 cell differentiation.

Analysis of liver lymphocytes from the relatively susceptible C57BL/6 mice revealed a pattern whereby the numbers of every investigated cell population increased slightly or significantly with infection by either *Ascaris* species. The opposite was true for the relatively resistant CBA/Ca mice, where all hepatic lymphoid cell numbers decreased with infection. This observation suggests, surprisingly, a more pronounced immune response to *Ascaris* by lymphoid cells in the susceptible C57BL/6 mice. It applies to B cells and conventional *αβ* T cells and their CD4^+^ and CD8^+^ T cell subsets, which can mediate pathogen-specific adaptive immunity *via* selective Th1/Th2/Th17 cytokine secretion and antibody production. It also applies to the innate lymphocytes, including *γδ* T cells, NK and NKT cells, which are uniquely abundant in the liver (Doherty, 2016).

NK cells, *γδ* T cells and NKT cells recognize conserved structures on pathogens and stressed host cells and respond rapidly by killing target cells and rapidly releasing cytokines. They account for the majority of lymphocytes in the liver and are thought to play roles in the initiation of immune responses against pathogens and tumours in an environment where immune tolerance is favoured over active immunity (Doherty, 2016). Little is known about the roles of these innate lymphocytes in *Ascaris* infection, but they are implicated in immunity against other parasites. *γδ* T cells are found in elevated numbers in the blood of patients with cutaneous leishmaniasis (Darabi *et al*., 2002), *Toxoplasma gondii* (Prigione *et al*., 2006) and *Schistosoma* infections (Schwartz *et al*., 2014) and in the mesenteric lymph nodes of *Schistosoma*-infected mice (Yu *et al*., 2014). *γδ* T cells numbers also increase after the acute phase of *Plasmodium* infection (Mamedov *et al*., 2018) and are required for the induction of immunity against *Plasmodium* in vaccination studies in mice (Zaidi *et al*., 2017). Subsets of NKT cells produce IFN-*γ* and IL-4 during egg deposition in the liver in mice infected with *S. mansoni* (Mallevaey *et al*., 2006; Mallevaey *et al*., 2007). Although these innate lymphocyte populations can selectively release Th2 cytokines and can polarize immune responses towards Th2, future studies are required to determine if any of these innate lymphocyte populations exhibit skewed Th2 phenotypes in response to *Ascaris* infection. Future studies are also required to explain why innate T cells exhibit opposite dynamics in the relatively susceptible and relatively resistant mouse strains in response to *Ascaris* infection.

In summary, our data demonstrate that infection with *A. suum* or *A. lumbricoides* results in increases in the numbers of myeloid cells, including monocytes, dendritic cells, Kupffer cells and eosinophils in the livers of both C57BL/6J and CBA/Ca mice, suggesting that these cells are likely to contribute to parasite elimination. Unexpectedly, the numbers of all hepatic lymphocyte subsets examined increased in the susceptible C57BL/6J mice but decreased in the relatively resistant CBA/Ca mice after infection with both parasite species, suggesting that the susceptible mice mounted more robust immune responses to the worms. This might be explained by the responses being more tolerogenic in C57BL/6J mice, leading to higher subsequent worm burdens in the lungs. Liver dendritic cells, Kupffer cells and monocytes are well-documented to preferentially induce tolerance over immunity to antigens encountered in the liver (Thomson and Knolle, 2010; Crispe, 2011; Doherty, 2016). Alternatively, the livers of the susceptible C57BL/6J mice may receive higher numbers of worms producing stimulatory antigens, requiring a more robust hepatic immune response compared with that of CBA/Ca mice. Consistent with the latter hypothesis, intestinal/rectal worm burdens are reported to be higher in C57BL/6J mice compared to CBA/Ca mice 6 days p.i. (Lewis *et al*., 2007), suggesting that predisposition may take place before the ascarid reaches the liver. Uninfected and *Ascaris*-infected CBA/Ca mice had higher numbers of CD4^+^ T cells and *γδ* T cells in their spleens which might arise from mesenteric lymph node activation by parasite antigens. Future functional studies are needed to elucidate the immunogenic *vs* tolerogenic roles of hepatic leucocytes in *Ascaris* infection.

## References

[ref1] Almeida S, Nejsum P and Williams AR (2018) Modulation of human macrophage activity by *Ascaris* antigens is dependent on macrophage polarization state. Immunobiology 223, 405–412. 29162324 10.1016/j.imbio.2017.11.003

[ref2] Anderson RM and May RM (1982) Population dynamics of human helminth infections: control by chemotherapy. Nature 297, 557–563. 7088139 10.1038/297557a0

[ref3] Arora P, Moll JM, Andersen D, Workman CT, Williams AR, Kristiansen K and Brix S (2020) Body fluid from the parasitic worm *Ascaris suum* inhibits broad-acting pro-inflammatory programs in dendritic cells. Immunology 159, 322–334. 31705653 10.1111/imm.13151 PMC7011627

[ref4] Bethony J, Brooker S, Albonico M, Geiger SM, Loukas A, Diemert D and Hotez PJ (2006) Soil-transmitted helminth infections: ascariasis, trichuriasis, and hookworm. Lancet (London, England) 367, 1521–1532. 16679166 10.1016/S0140-6736(06)68653-4

[ref5] Burnham KP and Anderson DR (2004) Multimodel inference. Sociological Methods & Research 33, 261–304.

[ref6] Coakley G, Volpe B, Bouchery T, Shah K, Butler A, Geldhof P, Hatherill M, Horsnell WGC, Esser-von Bieren J and Harris NL (2020) Immune serum-activated human macrophages coordinate with eosinophils to immobilize *Ascaris suum* larvae. Parasite Immunology 42, e12728. 32394439 10.1111/pim.12728

[ref7] Crispe IN (2011) Liver antigen-presenting cells. Journal of Hepatology 54, 357–365. 21084131 10.1016/j.jhep.2010.10.005 PMC3031082

[ref8] Croll NA and Ghadirian E (1981) Wormy persons: contributions to the nature and patterns of overdispersion with *Ascaris lumbricoides*, *Ancylosotma duodenale*, *Necator americanus* and *Trichuris trichiura*. Tropical and Geographical Medicine 33, 241–248. 7314236

[ref9] Croll NA, Anderson RM, Gyorkos TW and Ghadirian E (1982) The population biology and control of *Ascaris lumbricoides* in a rural community in Iran. Transactions of the Royal Society of Tropical Medicine and Hygiene 76, 187–197. 7101403 10.1016/0035-9203(82)90272-3

[ref10] Darabi H, Abolhassani M, Kariminia A and Alimohammadian MH (2002) Expansion of gammadelta T Cells in patients infected with cutaneous leishmaniasis with and without glucantime therapy. The Brazilian Journal of Infectious Diseases 6, 258–262. 12495608 10.1590/s1413-86702002000500007

[ref11] Deslyper G and Holland CV (2017) Overview on ascariasis in humans in South Asia. In Singh SK (ed.), Neglected Tropical Diseases-South Asia. Cham: Springer, pp. 83–120.

[ref12] Deslyper G, Colgan TJ, Cooper AJ, Holland CV and Carolan JC (2016) A proteomic investigation of hepatic resistance to *Ascaris* in a murine model. PLoS Neglected Tropical Diseases 10, e0004837. 27490109 10.1371/journal.pntd.0004837 PMC4974003

[ref13] Deslyper G, Doherty DG, Carolan JC and Holland CV (2019a) The role of the liver in the migration of parasites of global significance. Parasites & Vectors 12, 531. 31703729 10.1186/s13071-019-3791-2 PMC6842148

[ref14] Deslyper G, Holland CV, Colgan TJ and Carolan JC (2019b) The liver proteome in a mouse model for *Ascaris suum* resistance and susceptibility: evidence for an altered innate immune response. Parasites & Vectors 12, 402. 31412915 10.1186/s13071-019-3655-9 PMC6693097

[ref15] Deslyper G, Sowemimo OA, Beresford J and Holland CV (2020) *Ascaris lumbricoides* and *Ascaris suum* vary in their larval burden in a mouse model. Journal of Helminthology 94, e128. 32100653 10.1017/S0022149X20000127

[ref16] Doherty DG (2016) Immunity, tolerance and autoimmunity in the liver: a comprehensive review. Journal of Autoimmunity 66, 60–75. 26358406 10.1016/j.jaut.2015.08.020

[ref17] Dold C, Cassidy JP, Stafford P, Behnke JM and Holland CV (2010) Genetic influence on the kinetics and associated pathology of the early stage (intestinal-hepatic) migration of *Ascaris suum* in mice. Parasitology 137, 173–185. 19765333 10.1017/S0031182009990850

[ref18] Elkins DB, Haswell-Elkins M and Anderson RM (1986) The epidemiology and control of intestinal helminths in the Pulicat Lake region of Southern India. I. Study design and pre- and post-treatment observations on *Ascaris lumbricoides* infection. Transactions of the Royal Society of Tropical Medicine and Hygiene 80, 774–792. 3603617 10.1016/0035-9203(86)90384-6

[ref19] Enobe CS, Araújo CA, Perini A, Martins MA, Macedo MS and Macedo-Soares MF (2006) Early stages of *Ascaris suum* induce airway inflammation and hyperreactivity in a mouse model. Parasite Immunology 28, 453–461. 16916369 10.1111/j.1365-3024.2006.00892.x

[ref20] Favoretto BC, Silva SR, Jacysyn JF, Câmara NO and Faquim-Mauro EL (2014) TLR2- and 4-independent immunomodulatory effect of high molecular weight components from *Ascaris suum*. Molecular Immunology 58, 17–26. 24263181 10.1016/j.molimm.2013.10.011

[ref21] Gazzinelli-Guimarães PH, Gazzinelli-Guimarães AC, Silva FN, Mati VL, de Dhom-Lemos LC, Barbosa FS, Passos LS, Gaze S, Carneiro CM, Bartholomeu DC, Bueno LL and Fujiwara RT (2013) Parasitological and immunological aspects of early *Ascaris* spp. infection in mice. International Journal for Parasitology 43, 697–706. 23665127 10.1016/j.ijpara.2013.02.009

[ref22] Gazzinelli-Guimaraes PH, de Queiroz Prado R, Ricciardi A, Bonne-Année S, Sciurba J, Karmele EP, Fujiwara RT and Nutman TB (2019) Allergen presensitization drives an eosinophil-dependent arrest in lung-specific helminth development. Journal of Clinical Investigation 130, 3686–3701. 10.1172/JCI127963 PMC6715365 31380805

[ref23] Hamann KJ, Gleich GJ, Checkel JL, Loegering DA, McCall JW and Barker RL (1990) *In vitro* killing of microfilariae of *Brugia pahangi* and *Brugia malayi* by eosinophil granule proteins. Journal of Immunology 144, 3166–3173. 2324497

[ref24] Holland CV (2009) Predisposition to ascariasis: patterns, mechanisms and implications. Parasitology 136, 1537–1547. 19450374 10.1017/S0031182009005952

[ref25] Holland CV, Asaolu SO, Crompton DW, Stoddart RC, Macdonald R and Torimiro SE (1989) The epidemiology of *Ascaris lumbricoides* and other soil-transmitted helminths in primary school children from Ile-Ife, Nigeria. Parasitology 99(Pt 2), 275–285. 2594419 10.1017/s003118200005873x

[ref26] Holland CV, Behnke JM and Dold C (2013) Larval Ascariasis: impact, significance, and model organisms. In Holland C (ed.), Ascaris: The Neglected Parasite. Amsterdam: Elsevier, pp. 107–125.

[ref27] Hotez PJ, Aksoy S, Brindley PJ and Kamhawi S (2020) What constitutes a neglected tropical disease? PLoS Neglected Tropical Diseases 14, e0008001. 31999732 10.1371/journal.pntd.0008001 PMC6991948

[ref28] Javid G, Wani NA, Gulzar GM, Khan BA, Shah AH, Shah OJ and Khan M (1999) *Ascaris*-induced liver abscess. World Journal of Surgery 23, 1191–1194. 10501884 10.1007/s002689900645

[ref29] Lenth R (2019) emmeans: estimated marginal means, aka least-squares means. http://cran.r-project.org/package=emmeans/.

[ref30] Lewis R (2006) The development of a mouse model to explore resistance and susceptibility to early *Ascaris suum* infection. Ph.D. thesis. University of Dublin. 10.1017/S0031182005008978 16209722

[ref31] Lewis R, Behnke JM, Stafford P and Holland CV (2006) The development of a mouse model to explore resistance and susceptibility to early *Ascaris suum* infection. Parasitology 132, 289–300. 16209722 10.1017/S0031182005008978

[ref32] Lewis R, Behnke JM, Cassidy JP, Stafford P, Murray N and Holland CV (2007) The migration of *Ascaris suum* larvae, and the associated pulmonary inflammatory response in susceptible C57BL/6J and resistant CBA/Ca mice. Parasitology 134, 1301–1314. 17381887 10.1017/S0031182007002582

[ref33] Mallevaey T, Zanetta JP, Faveeuw C, Fontaine J, Maes E, Platt F, Capron M, de-Moraes ML and Trottein F (2006) Activation of invariant NKT cells by the helminth parasite *Schistosoma mansoni*. Journal of Immunology 176, 2476–2485. 10.4049/jimmunol.176.4.2476 16456008

[ref34] Mallevaey T, Fontaine J, Breuilh L, Paget C, Castro-Keller A, Vendeville C, Capron M, Leite-de-Moraes M, Trottein F and Faveeuw C (2007) Invariant and noninvariant natural killer T cells exert opposite regulatory functions on the immune response during murine schistosomiasis. Infection and Immunity 75, 2171–2180. 17353286 10.1128/IAI.01178-06 PMC1865739

[ref35] Mamedov MR, Scholzen A, Nair RV, Cumnock K, Kenkel JA, Oliveira JHM, Trujillo DL, Saligrama N, Zhang Y, Rubelt F, Schneider DS, Chien YH, Sauerwein RW and Davis MM (2018) A macrophage colony-stimulating-factor-producing *γδ* T cell subset prevents malarial parasitemic recurrence. Immunity 48, 350–363.e357. 29426701 10.1016/j.immuni.2018.01.009 PMC5956914

[ref36] Masure D, Vlaminck J, Wang T, Chiers K, Van den Broeck W, Vercruysse J and Geldhof P (2013) A role for eosinophils in the intestinal immunity against infective *Ascaris suum* larvae. PLoS Neglected Tropical Diseases 7, e2138. 23556022 10.1371/journal.pntd.0002138 PMC3605247

[ref37] McCallum HI (1990) Covariance in parasite burdens: the effect of predisposition to infection. Parasitology 100, 153–159. 2179831 10.1017/s0031182000060248

[ref38] McLaren DJ, Peterson CG and Venge P (1984) *Schistosoma mansoni*: further studies of the interaction between schistosomula and granulocyte-derived cationic proteins *in vitro*. Parasitology 88, 491–503. 6739134 10.1017/s0031182000054755

[ref39] McSharry C, Xia Y, Holland CV and Kennedy MW (1999) Natural immunity to *Ascaris lumbricoides* associated with immunoglobulin E antibody to ABA-1 allergen and inflammation indicators in children. Infection and Immunity 67, 484–489. 9916049 10.1128/iai.67.2.484-489.1999 PMC96345

[ref40] Midttun HLE, Acevedo N, Skallerup P, Almeida S, Skovgaard K, Andresen L, Skov S, Caraballo L, van Die I, Jørgensen CB, Fredholm M, Thamsborg SM, Nejsum P and Williams AR (2018) *Ascaris suum* infection downregulates inflammatory pathways in the pig intestine *in vivo* and in human dendritic cells *in vitro*. Journal of Infectious Diseases 217, 310–319. 29136163 10.1093/infdis/jix585

[ref41] Mitchell GF, Hogarth-Scott RS, Lewers HM, Edwards RD, Cousins G and Moore T (1976) Studies on immune response to parasite antigens in mice. I. *Ascaris suum* larvae numbers and antiphosphorylcholine responses in infected mice of various strains and hypothymic nu/nu mice. International Archives of Allergy and Immunology 56, 64–78. 10.1159/000231669 1087923

[ref42] Nogueira DS, Gazzinelli-Guimarães PH, Barbosa FS, Resende NM, Silva CC, de Oliveira LM, Amorim CC, Oliveira FM, Mattos MS, Kraemer LR, Caliari MV, Gaze S, Bueno LL, Russo RC and Fujiwara RT (2016) Multiple exposures to *Ascaris suum* induce tissue injury and mixed Th2/Th17 immune response in mice. PLoS Neglected Tropical Diseases 10, e0004382. 26814713 10.1371/journal.pntd.0004382 PMC4729520

[ref43] Prigione I, Chiesa S, Taverna P, Ceccarelli R, Frulio R, Morandi F, Bocca P, Cesbron-Delauw MF and Pistoia V (2006) T cell mediated immune responses to *Toxoplasma gondii* in pregnant women with primary toxoplasmosis. Microbes and Infection 8, 552–560. 16324868 10.1016/j.micinf.2005.08.008

[ref44] Pullan RL, Smith JL, Jasrasaria R and Brooker SJ (2014) Global numbers of infection and disease burden of soil transmitted helminth infections in 2010. Parasites & Vectors 7, 37. 24447578 10.1186/1756-3305-7-37 PMC3905661

[ref45] Reina Ortiz M, Schreiber F, Benitez S, Broncano N, Chico ME, Vaca M, Alexander N, Lewis DJ, Dougan G and Cooper PJ (2011) Effects of chronic ascariasis and trichuriasis on cytokine production and gene expression in human blood: a cross-sectional study. PLoS Neglected Tropical Diseases 5, e1157. 21666788 10.1371/journal.pntd.0001157 PMC3110165

[ref46] Ronéus O (1966) Studies on the aetiology and pathogenesis of white spots in the liver of pigs. Acta Veterinaria Scandinavica 7(suppl. 16), 11–112. 5949007

[ref47] Schwartz E, Rosenthal E and Bank I (2014) Gamma delta T cells in non-immune patients during primary schistosomal infection. Immunity, Inflammation and Disease 2, 56–61. 25400925 10.1002/iid3.18 PMC4220667

[ref48] Seo BS, Cho SY and Chai JY (1979) Frequency distribution of *Ascaris lumbricoides* in rural Koreans with special reference on the effect of changing endemicity. Kisaengch'unghak Chapchi. The Korean Journal of Parasitology 17, 105–113. 10.3347/kjp.1979.17.2.105 12902750

[ref49] Summan A, Nejsum P and Williams AR (2018) Modulation of human dendritic cell activity by *Giardia* and helminth antigens. Parasite Immunology 40, e12525. 29574798 10.1111/pim.12525

[ref50] Thomson AW and Knolle PA (2010) Antigen-presenting cell function in the tolerogenic liver environment. Nature Reviews Immunology 10, 753–766. 10.1038/nri2858 20972472

[ref51] Venables W and Ripley B (2002) Modern Applied Statistics, Fourth S., New York: Springer.

[ref52] Vlaminck J, Masure D, Wang T, Nejsum P, Hokke CH and Geldhof P (2016) A phosphorylcholine-containing glycolipid-like antigen present on the surface of infective stage larvae of *Ascaris* spp. is a major antibody target in infected pigs and humans. PLoS Neglected Tropical Diseases 10, e0005166. 27906979 10.1371/journal.pntd.0005166 PMC5131908

[ref53] Weatherhead JE, Porter P, Coffey A, Haydel D, Versteeg L, Zhan B, Gazzinelli Guimarães AC, Fujiwara R, Jaramillo AM, Bottazzi ME, Hotez PJ, Corry DB and Beaumier CM (2018) Larval infection and lung invasion directly induce severe allergic airway disease in mice. Infection and Immunity 86, e00533-18. 30249744 10.1128/IAI.00533-18 PMC6246907

[ref54] World Health Organization (2020) Neglected Tropical Diseases.

[ref55] Yu X, Luo X, Xie H, Chen D, Li L, Wu F, Wu C, Peng A and Huang J (2014) Characteristics of *γδ* T cells in *Schistosoma japonicum*-infected mouse mesenteric lymph nodes. Parasitology Research 113, 3393–3401. 24994455 10.1007/s00436-014-4004-8

[ref56] Zaidi I, Diallo H, Conteh S, Robbins Y, Kolasny J, Orr-Gonzalez S, Carter D, Butler B, Lambert L, Brickley E, Morrison R, Sissoko M, Healy SA, Sim BKL, Doumbo OK, Hoffman SL and Duffy PE (2017) Γ*δ* T Cells are required for the induction of sterile immunity during irradiated sporozoite vaccinations. Journal of Immunology 199, 3781–3788. 10.4049/jimmunol.1700314 PMC5698172 29079696

